# Evaluation of Surgical Cleavage Plane by Preoperative Magnetic Resonance Imaging Findings in Adult Intracranial Meningiomas

**DOI:** 10.3390/life12040473

**Published:** 2022-03-24

**Authors:** Nazmin Ahmed, Gianluca Ferini, Mosharef A. T. M. Hossain, Kanak Kanti Barua, Mohammad Nazrul Hossain, Giuseppe Emmanuele Umana, Nathan A. Shlobin, Gianluca Scalia, Paolo Palmisciano, Ottavio S. Tomasi, Bipin Chaurasia

**Affiliations:** 1 Department of Neurosurgery, Ibrahim Cardiac Hospital and Research Institute, Shahbag, Dhaka 1000, Bangladesh; nazmin.bsmmu@gmail.com (N.A.); drmnh2003@gmail.com (M.N.H.); 2 Department of Radiation Oncology, REM Radioterapia srl, 95029 Viagrande, Italy; gianluca.ferini@grupposamed.com; 3 Department of Neurosurgery, Bangabandhu Sheikh Mujib Medical University, Shahbag, Dhaka 1000, Bangladesh; hossain.mosharef@gmail.com; 4 School of Medicine, Bangabandhu Sheikh Mujib, Shahbag University, Dhaka 1000, Bangladesh; drbaruak@gmail.com; 5 Gamma Knife Center, Department of Neurosurgery, Cannizzaro Hospital, 95126 Catania, Italy; paolo.palmisciano94@gmail.com; 6 Department of Neurological Surgery, Feinberg School of Medicine, Northwestern University, Chicago, IL 60611, USA; nathan.shlobin@northwestern.edu; 7 Department of Neurosurgery, ARNAS Garibaldi, 95122 Catania, Italy; gianluca.scalia@outlook.it; 8 Department of Neurosurgery, Christian-Doppler-Klinik, Paracelsus Private Medical University, 5020 Salzburg, Austria; ottavio.tomasi@gmail.com; 9 Department of Neurosurgery, Neurosurgery Clinic, Birgunj 44300, Nepal; trozexa@gmail.com

**Keywords:** meningioma, surgical plane, cleavage, MRI, tumor size

## Abstract

(1) Background: Meningiomas are usually benign encapsulated intracranial tumors with well-defined borders that offer a high chance of cure with complete removal. The aim of this study was to evaluate the association between preoperative MRI features and surgical plane of cleavage. (2) Materials and Methods: This was a cross-sectional observational study conducted in the Department of Neurosurgery, Bangabandhu Sheikh Mujib Medical University. Data were recorded from 48 study participants with confirmed intracranial meningioma and analyzed by IBM SPSS (version 23). (3) Results: The highest incidence of intracranial meningioma was observed in the third decade of life of our study participants. The female to male ratio was 1.82:1. The most common location of meningiomas was convexity (29.16%), sphenoid wing (22.91%), parasagittal (20.83%), and falcine (14.58%). Most of the patients (68.8%) had medium-sized tumors, and 75.0% exhibited hyperintense signal change in the tumor parenchyma on T2-weighted imaging. One-third (33.3%) of patients were characterized as no edema, focal edema, and lobar/hemispheric edema. There was no statistically significant association between tumor size and types of surgical cleavage plane. Different signal intensities of tumor parenchyma, as well as types of peritumoral edema, showed no statistically significant association with surgical cleavage plane (*p* > 0.05). (4) Conclusion: There was no association among the size of the tumor, extent of peritumoral edema, the intensity of the tumor on T2-weighted images, and the types of surgical cleavage plane. Future studies with larger sample sizes are required to find out more precise findings.

## 1. Introduction

Meningiomas are the most common primary intracranial tumors, arising from the arachnoid cap cells and representing about 40% of symptomatic brain tumors in adults [1]. Their incidence increases with age, peaking after the fifth decade of life, and they affect females more commonly than males (2:1 ratio). The annual incidence per 100,000 population ranges from two to seven for females and one to five for males [2,3,4].

Standard brain MRI is the study of choice in the evaluation of intracranial neoplasms for diagnosis, surgical planning, and follow-up. MRI features of meningioma consist of homogenous enhancement, iso- to hypointensity on T1-weighted imaging (WI), hyperintensity on T2-WI, and the presence of a dural tail. The tumor grade can be predicted from the size of the tumor, presence of peritumoral edema, necrosis, and pattern of draining veins [5]. The extent of resection and histological grade are the most important factors related to progression free survival (PFS) after meningioma surgery [6,7,8]. The presence of a cleft sign on T2-WI and the absence of peritumoral edema are favorable factors for their resection [9].

Most meningiomas are benign and slowly growing tumors that are frequently curable after complete removal. Despite their benign histological and clinical nature, altered biological behavior is associated with locally aggressive behavior and late distant metastasis. Surgical treatment is usually recommended for patients with neurologic symptoms, large tumors, and/or associated cerebral edema. However, patients with more aggressive WHO II/III meningiomas may also benefit from early resection [5]. The general histological parameters that have been identified as indices of aggressive behavior and rapid recurrence of meningiomas are loss of architecture, cellularity, mitotic rate, diminution of nuclear/cytoplasmic ratio, foci of necrosis, nuclear pleomorphism, and adjacent brain parenchyma [10].

Tumor location, size, and histological features, including vascular and neural involvement, are important factors that determine the success of total surgical removal. To evaluate tumor-brain adhesions, including their location, Toaka et al. developed a novel imaging technique named brain surface motion imaging (BSMI), which is a method in which subtractions of images in the systolic and diastolic phases of cerebrospinal fluid (CSF)/brain pulsatile motion are created [9]. On the other hand, Slip Interface Imaging based on Magnetic Resonance Elastography, provides a method to non-invasively evaluate the degree of meningioma adhesion to adjacent brain tissue [11].

Primary studies focusing on the surgical cleavage plane of meningiomas are relatively uncommon. There is no primary research in Bangladesh focusing on this important association. The purpose of this study was to re-evaluate the relationship of tumor size, peritumoral edema, and signal intensity of tumor parenchyma with that of surgical cleavage plane for the first time in Bangladesh. The study also aimed to determine the association between intraoperative surgical cleavage plane types and preoperative MRI features of meningioma, which may play a significant role in surgical planning and preoperative counseling of the patient regarding the extent of surgically safe limit of tumor removal, particularly in eloquent areas, necessity of post-operative adjuvant therapy, tumor recurrence, and outcome.

## 2. Materials and Methods

This was a cross-sectional observational study in the Department of Neurosurgery of Bangabandhu Sheikh Mujib Medical University (BSMMU), Bangladesh, from March 2017 to March 2019. Patients with a confirmed intracranial meningioma by histopathology report, admitted in Department of Neurosurgery of BSMMU, who were aged more than 18 years, were included in the analysis. However, patients with recurrent meningiomas, multiple meningiomas, intraventricular meningiomas, cavernous sinus meningiomas, and pineal region meningiomas were excluded from the study given differences in presentation, preferred management/operative approach, or prognosis between these subtypes and standard meningiomas [12,13,14,15,16]. Patients with a previous history of whole brain radiation were also excluded from our study given secondary meningiomas following radiotherapy differ from primary meningiomas [17]. For example, the incidence of atypical or malignant meningiomas is twice as high for radiotherapy-induced meningiomas relative to spontaneous meningiomas. During the period of data collection, 48 patients fulfilled the selection criteria and were selected as study sample using a purposive sampling technique.

Before data collection, voluntary written informed consent was obtained from patients or legal guardians after complete explanation of the purpose of the study. During the admission, a detailed history was taken, and general and neurological examinations were also performed. Diagnosis of meningioma by MRI was made based on the intensity and enhancement features of the mass and the presence of ancillary findings including the dural tail sign and CSF cleft sign. The tumor size and intensity and peritumoral edema were noted. The surgical cleavage plane was observed and verified preoperatively by the chief neurosurgeon of different units in the institution. At the end of the procedure, histologically confirmed meningioma cases were ultimately selected for the study. The data collection sheet was designed by the researcher and approved by the institutional review board.

The data were entered in the data collection sheets and cleaned by the researcher using Microsoft Excel 2013 (Microsoft Inc., Remond, WA, USA). Data were processed and analyzed using the SPSS version 23 (IBM, Armonk, NY, USA) statistical package software. The results were described in frequencies and percentage. Statistical association among variables were investigated using the Chi-square test or Fisher’s exact test when appropriate. A *p*-value of < 0.05 was considered statistically significant.

Ethical clearance for the study was taken from the Department of Neurosurgery and Central Ethical Committee, BSMMU. The privacy of the patient was strictly maintained, and patient information was not disclosed to any source. Each of the steps of this study was completed in accordance with the Helsinki Declaration (1964).

### 2.1. Operational Definition

**Figure 1:**
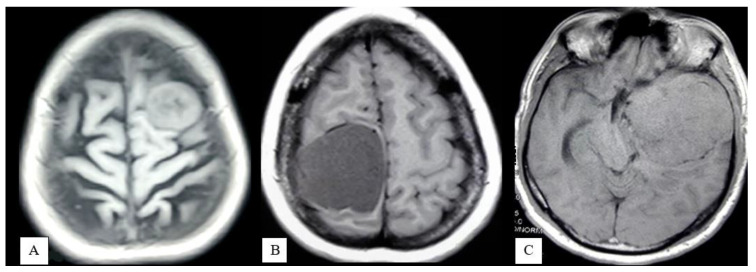
MRI of brain T1-WI axial section demonstrates small (**A**), medium (**B**), and large size meningioma (**C**).

Tumor Size: Tumor size was defined as the largest diameter, which was noted on the axial section of T1-weighted MR imaging sequences. The overall series was classified into three groups according to the size of the tumor:

1. Small (<3 cm) (Figure 1A);
2. Medium (3–6 cm) (Figure 1B);
3. Large (>6 cm) (Figure 1C).

**Figure 2:**
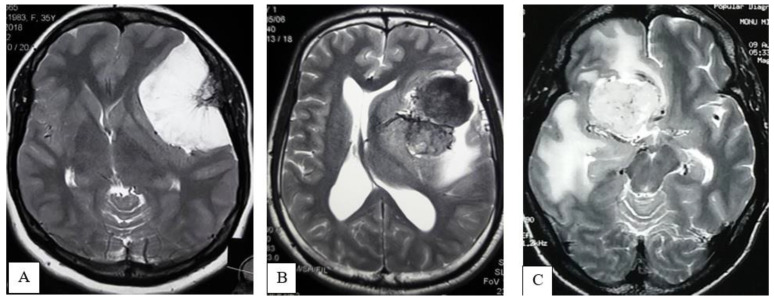
MRI of brain T2WI axial section demonstrates Absent peritumoral edema (**A**), Focal edema (**B**), Lobar or hemispheric edema (**C**).

Peritumoral Edema: Quantification of peritumoral edema was obtained from the axial section of T2-weighted MR imaging sequences and was graded as follows:

1. Absent: No evidence of peritumoral hyperintensity (Figure 2A);
2. Focal: Peritumoral hyperintensity is 3 cm or less in width (Figure 2B);
3. Lobar or hemispheric: Hyperintensity is more than 3 cm in width (Figure 2C).

Signal Intensity of the tumor parenchyma: The signal intensity of the tumor parenchyma was determined on T2-weighted MR imaging sequences and classified as

**Figure 3:**
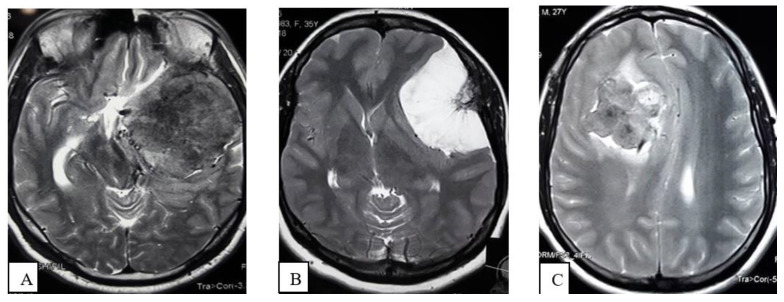
MRI of Brain, T2WI axial section demonstrates Iso- to hypointense (**A**), Hyperintense (**B**), Mixed intensity (**C**) tumor.

1. Iso-hypointense: Iso-hypointense to cortical gray matter (Figure 3A);
2. Hyperintense: Hyperintense to cortical gray matter (Figure 3B);
3. Mixed: Presence of hyperintense and iso-hypointense tumor parenchyma compared to cortical gray matter (Figure 3C).

### 2.2. Characterization of Surgical Cleavage Plane

**Figure 4:**
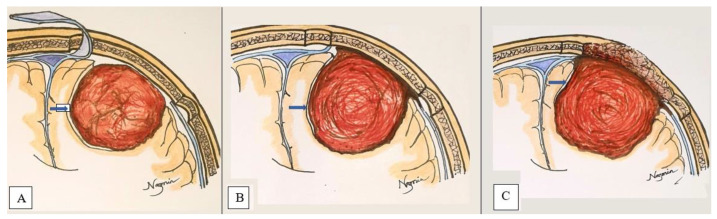
2-Dimensional schematic picture of a convexity meningioma demonstrates Extrapial (**A**), Mixed (**B**), and Subpial (**C**) surgical cleavage plane (marked by blue arrow).

Resection was performed by the chief neurosurgeon, who utilized a microsurgical dissection procedure and followed the four principles (dedressing, devascularization, debulking, dissection) of meningioma resection. The dissection plane was classified as follows:

1. Extrapial: Surgical cleavage plane lies outside the pia mater in more than two-thirds of the overall interface between tumor and cortex, regardless of whether an arachnoid membrane could be clearly identified (Figure 4A);
2. Mixed: Cleavage plane lies outside the pia mater in more than one-third but less than two-thirds of overall interface (Figure 4B);
3. Subpial: Tumor capsule exceeds the pia mater in more than two-thirds of the tumor cortex interface, and the surgeon was required to pass underneath the pia mater because of its incorporation into the tumor capsule (Figure 4C).

## 3. Results

The age distribution of 48 patients is shown in Table 1. The mean age of the patients was 43.58 ± 12.65 years (range 19–70 years). Most of the patients (27.1%) were between 31–40 years of age, followed by 51–60 years (25.0%). The female to male ratio was 1:1.82.

Most of the meningiomas were convexity (29.16%) followed by sphenoid wing (22.91%), parasagittal (20.83%), and falcine meningiomas (14.58%). More than 12% were also seen in other locations such, as the orbital roof, planum sphenoidale, cerebello-pontine angle, and middle cranial fossa. Most patients (68.8%) had medium size tumors, 20.8% had large size tumors, and only 10.4% had small tumors. Thirty-six patients (75.0%) exhibited hyperintense signal change in the tumor parenchyma on T2-WI, nine patients (18.8%) showed iso- to hypointense signal change, and three patients (6.3%) showed mixed intensity. One-third (33.3%) of patients were characterized as no edema, focal edema, and lobar/hemispheric edema.

Thirty patients (62.5%) underwent Simpson grade II resection, 15 patients (31.3%) underwent grade I resection, and the remaining three patients (6.2%) underwent grade 0 resection. When looking at histopathological findings, 97.9% was diagnosed as WHO grade I meningioma, and only 2.1% were diagnosed as WHO grade II (Table 1).

During evaluation of the surgical cleavage plane, there was no statistically significant association observed between tumor size and types of surgical cleavage plane (*p* < 0.05). Different signal intensity of tumor parenchyma, as well as types of peritumoral edema, also showed no statistically significant association (*p* < 0.05) with the surgical cleavage plane (Table 2).

## 4. Discussion

This is the first study in Bangladesh to assess intracranial meningiomas in adults and evaluate the surgical cleavage plane through preoperative MRI findings. Moreover, the association between characteristics of the tumor via preoperative MRI features and surgical cleavage plane was analyzed. A high percentage of recurrence of intracranial meningiomas has been reported in various studies due to primary failure of complete microsurgical resection of the lesions [2]. An inappropriate or altered tumor cleavage plane is another important factor that predicts unfavorable surgical outcomes [18].

Our analysis showed a high proportion of convexity meningiomas followed by sphenoid wing and parasagittal meningiomas. These findings are like the study conducted by Moradi et al. in 2008 [19]. The MRI appearance of the intracranial meningioma and its usefulness in assessing tumor location, vascularity, neurovascular encasement, and venous sinus invasion has been the subject of several articles [20,21]. In keeping with previous studies, we examined tumor size, signal intensity of tumor parenchyma on T2-WI, and peritumoral edema [22,23].

We observed no statistically significant association between tumor size and surgical plane of cleavage. The results indicate that evaluation of the surgical cleavage plane during the operation based on tumor size alone was difficult. Similarly, Takeguchi et al. observed no correlation between size of tumor and degree of adhesion [24]. On the other hand, Thenier-villa et al. did not find a relationship between tumor volume and surgical plane of cleavage. However, tumor size reflects is a proxy for how challenging a procedure is in terms of duration and risk of perioperative hemorrhage [18].

The signal intensity of the meningioma parenchyma obtained by T2-WI reflected cellularity, fiber components, calcification, and tumor consistency [25]. According to some previous studies, the degree of tumor brain adhesion is low in iso- to hypointense tumors, while the degree of adhesion tended to be high in hyperintense tumors [26]. The study conducted by Takeguchi et al. observed no significant difference in the degree of adhesion by the intensity of tumor parenchyma obtained in T2-WI [24]. In the present study, no statistically significant association was observed between the surgical plane of cleavage and signal intensities of tumor parenchyma. Preoperative verification of consistency was challenging because of the absence of a cavitron ultrasound aspirator. Additionally, all interpretation was conducted by one neurosurgeon, perhaps adding bias.

Few studies in the literature have addressed the direct relationship between peritumoral edema and the surgical cleavage plane during surgery [17,21,25]. Salpietro et al. reported that the degree of peritumoral edema was correlated with cortical penetration in patients with meningioma [26]. Thenier-villa et al. studied 83 convexity meningiomas and concluded that peritumoral edema was an independent predictor of the altered cleavage plane [18]. However, in this study we found no significant association between the types of peritumoral edema and surgical cleavage plane. In our hospital set-up, some of our patients received steroid preoperatively to maximize the neurological outcome and reduce peritumoral edema. An inability to control for this variable is likely an important reason preventing us from establishing an association between peritumoral edema and types of surgical plane of cleavage, which aligns with previous studies [27,28].

In our study, three patients (6.2%) underwent Simpson grade 0 resection, 15 patients (31.3%) underwent grade I resection, and the remaining 30 patients (62.5%) underwent grade II resection, consistent with the previous studies [21,25,29,30,31,32]. Pavelin et al. observed that, about 80% meningiomas are slow-growing tumors of WHO grade I, and the most diagnosed histological subtypes are meningothelial, fibrous and transitional meningioma [25]. Our analysis also observed a high proportion of WHO grade I tumors. Future studies with a large sample size may provide greater variation in histopathological findings and allow determination of the influence of surgical cleavage plane.

### Limitations of the Study

The study was conducted with several limitations. The limited sample size may have reduced the ability to detect an association between tumor size and surgical plane of cleavage. Although findings on MRI were analyzed carefully with MRI reports and finally verified by professional expert, this study may have underestimated interobserver variability. This study occurred at a single center, subjecting the results to the management preferences of that single neurosurgeon who performed the resections.

## 5. Conclusions

Presurgical evaluation of adhesion between the brain and meningioma is advantageous for surgical planning of meningioma cleavage. Our analysis showed that there was no statistically significant association between the size of the tumor, extent of peritumoral edema, intensity of the tumor on T2-weighted MR images, and types of surgical cleavage plane. Smaller tumors are easier to locate in the extrapial plane of cleavage. We emphasize that early removal of meningiomas—prior to symptomatic manifestation—is preferred. Adjuvant treatment with steroids should be utilized to reduce peritumoral edema.

## Figures and Tables

**Table 1:** Descriptive statistics of the patient demographics.

Variables	Category	Frequency (n)	Percentage (%)
Age	≤30	10	20.8
	31–40	13	27.1
	41–50	10	20.8
	51–60	12	25.0
	>60	3	6.3
Gender	Male	31	64.6
	Female	17	35.4
Location of meningioma	Convexity	14	29.16
	Sphenoid wing	11	22.91
	Parasagittal	10	20.83
	Falcine	7	14.58
	Others	6	12.5
Tumor size	Small	5	10.4
	Medium	33	68.8
	Large	10	20.8
Tumor intensity	Hyperintense	36	75.0
	Iso- to hypointense	9	18.8
	Mixed	3	6.3
Peritumoral edema	Focal edema	16	33.3
	Lobar or hemispheric edema	16	33.3
	No edema	16	33.3
Simpson’s grading	Grade 0	3	6.2%
	Grade I	15	31.5
	Grade II	30	62.5
Histopathological findings	Meningioma, WHO—I	47	97.9
	Meningioma, WHO—II	1	2.1

**Table 2:** Association between tumor characteristics and surgical plane of cleavage.

Variables	Category	Surgical Plane of Cleavage			*p* Value
		Extrapial (%)	Mixed (%)	Subpial (%)	
Tumor size	Small	4 (80.0)	1 (20.0)	0 (0.0)	0.055
	Medium	17 (81.8)	3 (9.1)	3 (9.1)	
	Large	5 (50.0)	5 (50.0)	0 (0.0)	
Intensity of tumor parenchyma	Hyperintense	28 (77.8)	6 (16.7)	2 (5.6)	0.881
	Iso- to hypointense	6 (66.7)	2 (22.2)	1 (11.1)	
	Mixed	2 (66.7)	1 (33.3)	0 (0.0)	
Peritumoral edema	No edema	12 (75.0)	3 (18.8)	1 (6.3)	0.934
	Focal	13 (81.3)	2 (12.5)	1 (6.3)	
	Lobar or hemispheric	11 (68.8)	4 (25.0)	1 (6.3)	

## Data Availability

Not applicable.
